# Functional Characterization of the SHIP1-Domains Regarding Their Contribution to Inositol 5-Phosphatase Activity

**DOI:** 10.3390/biom15010105

**Published:** 2025-01-10

**Authors:** Spike Murphy Müller, Nina Nelson, Manfred Jücker

**Affiliations:** Institute of Biochemistry and Signal Transduction, University Medical Center Hamburg-Eppendorf, 20246 Hamburg, Germany; spike.mueller@stud.uke.uni-hamburg.de (S.M.M.); nina.nelson@mbg.au.dk (N.N.)

**Keywords:** SHIP1, phosphatidylinositol phosphatase, phosphatidylinositide 3-kinase (PI 3-kinase), AKT (=PKB), PI3K/AKT signaling pathway, phosphatase activity, enzyme catalysis, enzyme kinetics, allosteric regulation, enzyme regulation, polymerase chain reaction (PCR), cloning, protein expression, Western blot

## Abstract

The Src homology 2 domain-containing inositol 5-phosphatase 1 (SHIP1) is a multidomain protein consisting of two protein–protein interaction domains, the Src homology 2 (SH2) domain, and the proline-rich region (PRR), as well as three phosphoinositide-binding domains, the pleckstrin homology-like (PHL) domain, the 5-phosphatase (5PPase) domain, and the C2 domain. SHIP1 is commonly known for its involvement in the regulation of the PI3K/AKT signaling pathway by dephosphorylation of phosphatidylinositol-3,4,5-trisphosphate (PtdIns(3,4,5)P_3_) at the D5 position of the inositol ring. However, the functional role of each domain of SHIP1 for the regulation of its enzymatic activity is not well understood. To determine the contribution of the individual domains to catalytic activity, the full-length protein was compared with truncated constructs lacking one or more domain(s), regarding the substrate turnover (k_cat_) and catalytic efficiency (k_cat_/K_m_) towards ci8-PtdIns(3,4,5)P_3_. With this approach, it was possible to verify the allosteric activation of SHIP1 mediated by the C2 domain as described previously, while the PHL domain seemed instead to have a negative effect regarding catalytic efficiency. The full-length SHIP1 clearly displayed the highest turnover and the second-highest catalytic efficiency, showing the role of the SH2 domain and PRR not only in protein–protein interactions but also in catalysis. The SH2 domain increased substrate turnover but negatively affected catalytic efficiency. The linker between the SH2 and the PHL domains decreased the turnover number but positively influenced the catalytic efficiency. The PRR increased both the substrate turnover and the protein’s catalytic efficiency. The regression analysis of the Michaelis–Menten graph revealed SHIP1 to be an allosteric enzyme, with the PRR and the linker being the most involved domains in that regard. In summary, our data indicate a complex regulation of the enzymatic activity of SHIP1 by its individual domains. While the C2 domain and PRR at the carboxy-terminus have a positive effect on enzymatic activity, the SH2 and PHL domain at the amino-terminus inhibit catalytic efficiency.

## 1. Introduction

The phosphoinositide-5-phosphatase 1 (SHIP1) is predominantly expressed in hematopoietic cells [1], where it was shown to be essential for the regulation of their survival and proliferation [2]. This primary function, although not the only one, could be attributed for the most part to the downregulation of the phosphatidylinositol-3,4,5-trisphosphate PtdIns(3,4,5)P_3_ content of the cell, which reduces the activation of the protein kinase B (AKT) [2]. Low SHIP1 activity was associated with acute myeloid leukemia (AML) [3], acute lymphoblastic leukemia (ALL) [4], and chronic myeloid leukemia (CML) [5]. Additionally, SHIP1 may be involved in the emergence of non-hematopoietic types of cancer, such as colorectal cancer [6], non-small-cell lung cancer (NSCLC) [7], and pancreatic cancer [8]. Furthermore, a link between SHIP1 deficiency and inflammatory bowel diseases (IBD), such as Crohn’s disease (CD) [9], as well as Japanese encephalitis virus (JEV)-induced neuroinflammatory processes [10] and *Trichuris muris* infections [11], has been suggested. Lastly, involvement as a possible risk factor for the development of late-onset Alzheimer’s disease (LOAD) [12] and acute ischemic stroke (AIS) in Chinese patients [13] has been proposed in the literature. Overall, it seems that knowledge of the exact function and structure of the SHIP1 protein will become important in the future when looking for possible therapeutic targets in the treatment of those diseases. It is known so far that SHIP1 displays a structure of five domains, i.e., the Src homology two (SH2) domain, the pleckstrin homology-like (PHL) domain, the inositol 5-phosphatase (5PPase) domain, the C2 domain, and the proline-rich region (PRR) [2]. The SH2 domain was shown to mediate interactions with phosphotyrosines following the activation of upstream receptors with the help of its FLVR motif [14]. The PHL domain was found to bind PtdIns(3,4,5)P_3_, a substrate of SHIP1 [15]. This was shown to mediate the directed recruitment of SHIP1 to the membrane of phagocytic cups of macrophages following their stimulation with IgG-opsonized beads, which resulted in the inhibition of phagocytosis [15]. The 5PPase domain is required for catalysis and contributes to rather unspecific initial membrane association via its membrane interaction motif (MIM) [16]. The C2 domain was demonstrated to be an allosteric activator of SHIP1 via association with the product phosphatidylinositol-3,4-bisphosphate (PtdIns(3,4)P_2_) [17]. The PRR domain mediates interaction with Src homology 3 (SH3) domain-containing proteins via its PXXP motifs, as well as phosphotyrosine-binding (PTB) domain-containing ones via its NPXY motifs [14]. So far, the specific function and interplay of the individual domains with regard to catalysis have not been clarified. In this study, we aimed to determine the catalytic efficiency of full-length SHIP1 (FL), as well as several constructs with one or more domains truncated in order to clarify the individual contribution of each SHIP1 domain to catalysis.

## 2. Materials and Methods

### 2.1. Generation of SHIP1 Sequence-Containing Expression Vectors

**Figure 1:**
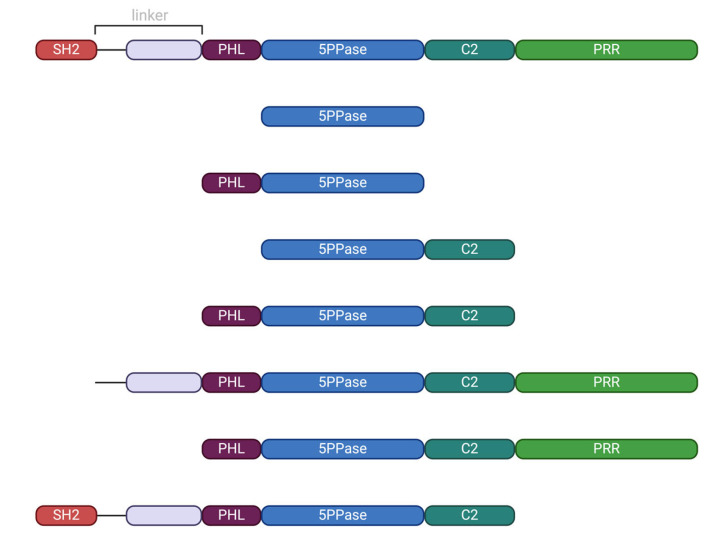
SHIP1 Domain Constructs [Created in BioRender. Müller, S. (2024) https://BioRender.com/r91z694, (accessed on 1 November 2024)]. The domain lengths in this figure are scaled to match the lengths of the corresponding amino acid sequence. From top to bottom: SHIP1 FL, 5PPase, PHL-5PPase, 5PPase-C2, PHL-5PPase-C2, linker-PHL-5PPase-C2-PRR (ΔSH2), PHL-5PPase-C2-PRR (ΔSH2-linker), SH2-linker-PHL-5PPase-C2 (ΔPRR).

The desired parts of the SHIP1 gene sequence were amplified in a polymerase chain reaction (PCR) using the primers (Eurofins Genomics, Ebersberg, Germany) listed below (Table 1), thereby creating versions lacking the sequence for one or more of the domains (Figure 1). The PCR was carried out using the Phusion™ Hot Start II DNA polymerase (ThermoFisher Scientific, Dreieich, Germany) with an initial denaturation for 30 s at 98 °C followed by 20 cycles of denaturation for 10 s at 98 °C, primer annealing for 30 s at 6 °C below the lowest melting temperature (T_m_), and elongation at 72 °C for 30 s per 1000 base pairs (kb) followed by a final incubation for eight minutes at 72 °C. Those DNA constructs were cloned into pASG-IBA5 (IBA Lifescience, Biozol, Eching, Germany) using the IBA Lifescience StarGate^®^ cloning system as described by the manufacturer. Briefly, the primers contained restriction and recognition sites to allow for the cloning of the PCR products into the entry vector, pENTRY-IBA51 (IBA Lifescience, distributed by Biozol, Eching, Germany), using *Lgu*I (ThermoFisher Scientific, Dreieich, Germany), and the resulting donor vector contained restriction and recognition sites to allow for the cloning of the previously inserted DNA into the acceptor vector, pASG-IBA5, using *Esp*3I (ThermoFisher Scientific, Dreieich, Germany), thereby generating the expression vector. The entry vector carries a kanamycin resistance cassette for antibiotic selection. The acceptor vector carries an ampicillin resistance cassette for antibiotic selection and a tet-on system for tetracycline-controlled transcriptional activation. The acceptor vector further contains the sequence of a Strep-tag^®^ II between the start codon and the gene of interest (GOI) to allow for the expression of N-terminally Strep-tagged fusion proteins. After the purification of the expression vector, the sequence was checked via enzymatic restriction and gel electrophoresis, as well as subsequent forward and reverse sequencing.

### 2.2. Expression and Purification of SHIP1 Domain Constructs

SHIP1 expression and purification was performed as described previously [18,19]. In short, the SHIP1 DNA construct-containing vectors were subsequently transformed into *E. coli* BL21(DE3)pLysSpREP4 (ThermoFisher Scientific, Dreieich, Germany). The main culture was inoculated with a pre-culture and allowed to grow until an OD_600_ of 0.6 was reached. Expression was induced by the addition of 200 ng/mL anhydrotetracycline, and the culture was incubated for 3 h at room temperature (RT) or 22 h at 4 °C. Bacteria were harvested by centrifugation at approximately 4000 g and resuspended in buffer W (IBA Lifesciences, distributed by Biozol, Eching, Germany) containing 100 mM Tris, 150 mM sodium chloride (NaCl), and 1 mM ethylenediaminetetraacetic acid (EDTA) at a pH of 8.0, supplemented with 0.5% Triton X-100, 1 mM Dithiothreitol (DTT), 1 mM benzamidine, and 1 mM phenylmethylsulfonyl fluoride (PMSF). They were lysed by ultrasonication in three 45 s intervals at 90% constant intensity, and the lysate was centrifuged at 16,000× *g* for 30 min to remove cellular debris. The lysate was loaded onto a StrepTactin^®^XT Superflow^®^ gravity flow resin (IBA Lifesciences, distributed by Biozol, Eching, Germany), equilibrated with 2 column bed volumes (CV) buffer W (IBA), and allowed to flow through at a flow rate of 1 mL/minute. The resin was washed with 8 CV buffer W and eluted in three steps using 0.6 CV (E1), 1.6 CV (E2), and 0.8 CV (E3) buffer BXT (IBA Lifesciences, distributed by Biozol, Eching, Germany) containing 100 mM Tris, 150 mM NaCl, 1 mM EDTA, and 50 mM biotin at a pH of 8.0. The resin was regenerated using 10–20 CV 20 mM NaOH followed immediately by 10–20 CV buffer W. The elution fractions were analyzed by SDS-PAGE and Coomassie staining as well as Western blot using the Strep-MAB Classic HRP antibody (IBA Lifesciences, distributed by Biozol, Eching, Germany) and the SuperSignal™ West Pico PLUS Chemiluminescent Substrate (ThermoFisher Scientific, Dreieich, Germany), as indicated by the manufacturers. Those samples containing SHIP1 were pooled and shock-frozen in liquid nitrogen in 10–20 µL aliquots. The concentration was determined densitometrically in the Coomassie-stained gels using a BSA standard. Eventually, elution fractions were concentrated using the Amicon^®^ 100 K ultrafiltration system (Sigma-Aldrich, distributed by Merck, Taufkirchen, Germany) (Table 2).

### 2.3. Phosphatase Assay of SHIP1 Domain Constructs

The enzymatic activity was determined by a malachite-green-based phosphatase assay, which detects the phosphate liberated in the phosphatase reaction, as described previously [20]. In brief, SHIP1 constructs were diluted to a final concentration of 5 ng/µL in phosphatase assay buffer containing 20 mM triethanolamine (TEA), 100 mM potassium chloride (KCl), and 2.5 mM magnesium chloride (MgCl_2_) at a pH of 7.2. The reaction mix was pre-incubated for 5 min at 37 °C. The substrate PtdIns(3,4,5)P_3_ diC8 (P-3908, Echelon Bioscience, Salt Lake City, UT, USA) was added at concentrations of 0, 5, 10, 20, 50, 100, and 300 µM. After substrate addition, 90 µL samples were taken at 5, 20, 45, and 60 s, and the reactions were stopped by the addition of 31.5 µL 0.1 M EDTA. In total, 80 µL of the stopped reaction was mixed with 20 µL of the phosphatase assay working solution (POMG-25H, BioAssay Systems, distributed by Biozol, Eching, Germany) and incubated for 30 min at RT. Subsequently, the amount of phosphate liberated by the phosphatase reaction was measured at 620 nm in a microplate reader and quantified using a sodium dihydrogen phosphate (NaH_2_PO_4_) standard curve. The dilution factor of 1.3 due to the addition of EDTA was taken into account. Each measurement for the phosphate standard and the activity of the constructs was examined in at least threefold determination. From the experiments, the specific enzyme activity of the domain constructs in U/mg, in the form of the initial reaction velocity, was determined and plotted against the concentration of the substrate. The V_max_ and K_m_ values were calculated with the GraphPad Prism program using sigmoidal or hyperbolic regression. The concentration of SHIP1 in the assay was 5 ng/µL, with the final volume in the microwell plate being 80 µL. A factor of 1.3 was applied to take into account the dilution from the addition of EDTA. To calculate the amount of SHIP1 domain constructs in moles, the molecular weight was determined using the ExPASy Compute Pi/Mw tool (Table 3). For the full-length protein, an established molar mass of 145 kDa was used. The turnover number k_cat_ was then determined by using the formula k_cat_ = V_max_/E_0_, and the catalytic efficiency was k_cat_/K_m_.

## 3. Results

### 3.1. Expression and Purification of SHIP1 Domain Constructs

**Figure 2:**
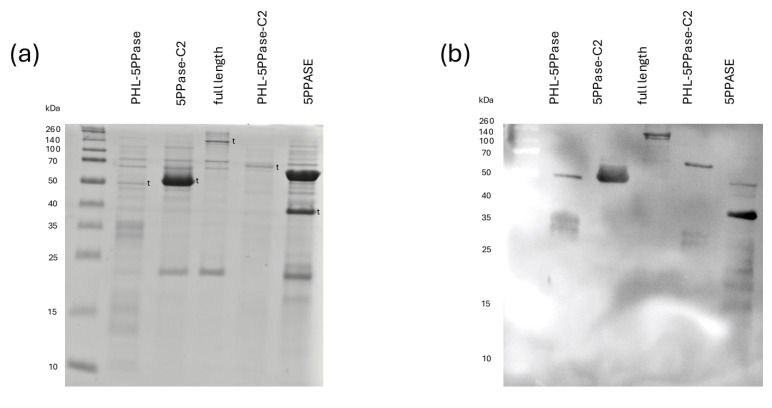

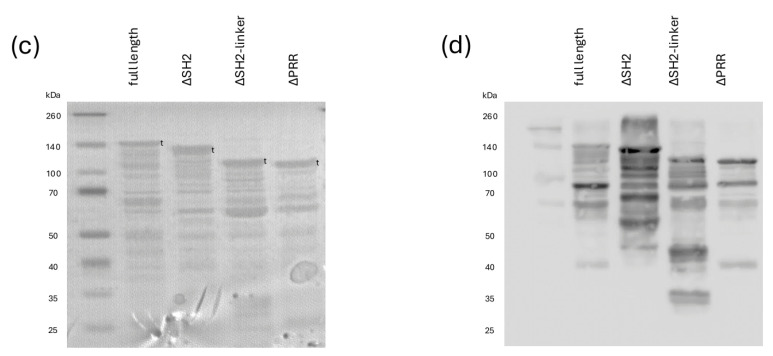
SDS-PAGE and Western blot (**a**,**c**) show the Coomassie-stained SDS-PAGE gel, and (**b**,**d**) the Western blot results with the Strep-MAB Classic HRP conjugate antibody, according to Section 2.2, after the expression and purification of Strep-tagged SHIP1 domain constructs. Target bands were marked with a t to the right of the band. The following constructs are shown from left to right: (**a**,**b**) PHL-5PPase; 5PPase-C2; FL; PHL-5PPase-C2; 5PPase; (**c**,**d**) FL; linker-PHL-5PPase-C2-PRR (ΔSH2); PHL-5PPase-C2-PRR (ΔSH2-linker); SH2-Linker-PHL-5PPase-C2 (ΔPRR).

The presence of proteins with the same sizes as the desired SHIP1 domain constructs (Figure 1) could be confirmed by Coomassie staining following SDS-PAGE (Figure 2). Densometric protein determination in the Coomassie-stained gel, using a BSA standard, showed a protein yield ranging between 260 and 1190.25 ng/µL (Table 2). The samples were further analyzed by Western blot using the N-termianal Strep-Tag^®^ II as a target for the Strep-MAB Classic antibody with a HRP conjugate (Figure 2b), which confirmed the assumption that the detected proteins were the SHIP1 domain constructs.

### 3.2. Phosphatase Assay of SHIP1 Domain Constructs

**Figure 3:**
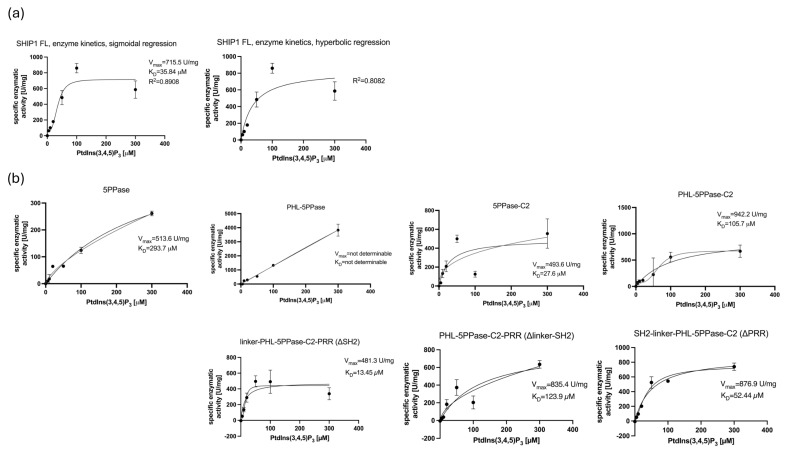
Curves of the Michaelis–Menten Equation. (**a**) Determination of the enzyme kinetics of SHIP1 FL towards PtdIns(3,4,5)P_3_. The specific activity of SHIP1 FL was determined for substrate concentrations ranging from 0 to 300 µM, plotted against the substrate concentration in µM and analyzed by hyperbolic or sigmoidal regression to determine the V_max_ and K_m_. Error bars indicate standard deviations. *n* = 3. (**b**) Determination of the enzyme kinetics of SHIP1 5PPase, SHIP1 PHL-5PPase, SHIP1 5PPase-C2, SHIP1 PHL-5PPase-C2, SHIP1 linker-PHL-5PPase-C2-PRR (ΔSH2), SHIP1 PHL-5PPase-C2-PRR (ΔSH2-linker), and SHIP1 SH2-linker-PHL-5PPase-C2 (ΔPRR) domain constructs towards PtdIns(3,4,5)P_3_. The specific activity of the constructs was determined for substrate concentrations ranging from 0 to 300 µM, plotted against the substrate concentration in µM and analyzed by hyperbolic or sigmoidal regression to determine the V_max_ and K_m_. Error bars indicate standard deviations. *n* =3.

The specific enzyme activity at different substrate concentrations was determined for the full-length SHIP1 protein and seven SHIP1 domain constructs (Figure 3), and they were analyzed concerning their maximum reaction velocity V_max_, their Michaelis constant K_m_, their catalytic constant k_cat_, also known as turnover number, and their specificity constant k_cat_/K_m_, also known as catalytic efficiency. Most notably, the full-length construct showed the highest turnover number (Table 4), and the ΔSH2 construct showed the highest catalytic efficiency (Table 4). The ΔPRR and ΔSH2 constructs had less overall substrate turnover than the full-length construct, and the ΔSH2-linker construct showed a higher turnover than the ΔSH2 construct (Table 4). The 5PPase-C2 construct showed a higher turnover than the 5PPase alone, and the addition of the PHL domain increased the turnover further (Table 4). In detail, when comparing the SHIP1 FL and the SHIP1 PHL-5PPase-C2 construct missing the SH2 domain and PRR, the loss of the domains is shown to decrease the catalytic efficiency as well as the turnover number by factors of 0.203 and 0.6. The comparison of the SHIP1 FL and the SHIP1 ΔSH2, to elucidate from which domain this effect resulted, shows that the SH2 domain increased the turnover by a factor of 1.748 and decreased the catalytic efficiency by a factor of 0.656. When looking at the difference in activity between SHIP1 FL and SHIP1 ΔPRR, an increase in both of those parameters is shown with the factors of 1.187 for the turnover and of 1.737 for the efficiency. The PHL increased the turnover by a factor of 2.4 and decreased the efficiency by a factor of 0.627, when comparing the 5PPase-C2 and PHL-5PPase-C2 constructs. The linker between the SH2 domain and the PHL domain inversely altered the enzyme activity. Between the ΔSH2-linker and ΔSH2 constructs, there was an increase in catalytic efficiency by a factor of 6.402 when adding the linker to the construct and a decrease in substrate turnover by a factor of 0.695. The K_m_ was reduced by a factor of 0.109 when adding the linker to the protein.

Comparing the adherence towards the two regression models (Table 5), hyperbolic and sigmoidal regression, we found that SHIP1 FL followed the sigmoidal regression 8.26% better and the ΔSH2 construct followed the sigmoidal regression 8.5% better than the hyperbolic regression. Deleting the linker decreased adherence towards the hyperbolic regression but still resulted in a 2.46% better adherence to the sigmoidal regression over the hyperbolic regression. Truncation of the PRR highly reduced the delta between adherence to the sigmoidal and hyperbolic regression and resulted in nearly equal adherence to both models, with only 0.98% better adherence to the sigmoidal regression. The truncation of the PHL and the truncation of the C2 domains both reduced the delta of the adherence towards the sigmoidal regression minus the hyperbolic regression. In general, all constructs and the SHIP1 FL curves matched the sigmoidal regression curve better than the hyperbolic regression curve.

## 4. Discussion

In this study, we determined the catalytic properties of constructs bearing individual or combined SHIP1 domains. Our aim was to elucidate the individual contribution of each SHIP1 domain to catalysis. For that purpose, we used both the substrate turnover k_cat_ and the catalytic efficiency k_cat_/K_m_ in the comparison of the constructs. The turnover represents the maximum capacity of the enzyme with respect to substrate concentration [21]. Most enzymes have been reported to process about 1 to 10,000 molecules per second; however, turnover numbers of up to 40,000,000 per second have been reported for catalase [22]. Other than the V_max_, k_cat_ is not dependent on the amount of protein added to a reaction but rather the amount of active enzyme sites and therefore allows for the comparison independent of the mass of a protein. The catalytic efficiency, however, is the most often used catalytic parameter. Using the K_m_ value in its calculation allows for the analysis of the behavior at different substrate concentrations. The lower the K_m_, the higher the affinity and the higher the reaction velocity at lower substrate concentrations [21]. However, to give a full and differentiated overview of our findings, we will be looking at both those numbers. In general it can be mentioned that catalytic efficiency is more important for interpretations of the possible physiological function, if the physiological concentration of PtdIns(3,4,5)P_3_ is lower than necessary to demand the maximum capacity of the protein. However, the relevant exact concentration in living cells is not known and depends greatly on the activity status of the cell and the area that is looked at. The activation of the cell via growth factors, cytokines, insulin, and RAS-stimulating agents would result in the generation of PtdIns(3,4,5)P_3_ [23] and therefore in the increase in its concentration. An increase in PtdIns(3,4,5)P_3_ concentration is also known for the activation of the PI3-Kinase [24]. Furthermore, PtdIns(3,4,5)P_3_ is, other than in our experiment, membrane-bound via its acyl chain [25]. According to the lipid rafts model [26], it is possible that PtdIns(3,4,5)P_3_ is highly concentrated at specific membrane regions when the cell is stimulated. We do not know if the conditions in the living human cell require the maximum performance of the enzyme, which is best described by the k_cat_ value, or if the substrate concentration is as low as to not demand the full capacity, for which the k_cat_/K_m_ would be a better indicator. Optimally, the substrate concentrations for the assay would be chosen to represent the physiological concentrations. This was not possible in this case, because the assay was performed in a solution with soluble protein. The affinity for the protein towards the substrate is therefore probably lower because there is less spatial proximity between the two.

Summarizing our results, we were able to verify that the PRR as well as the C2 domain increase both substrate turnover and catalytic efficiency, which is in concordance with two other recent studies [27,28]. The SH2 domain increased turnover but negatively affected catalytic efficiency. This is mathematically due to a disproportionate reduction in the K_m_ compared to the reduction in the V_max_ for the ΔSH2 construct, indicating that the SH2 domain is more relevant for the activity of SHIP1 at higher substrate concentrations. A definitive explanation for this finding cannot be provided. Several theories are possible. For example, the substrate could function as an allosteric activator at the SH2 domain but bind with a low affinity in this region. In this case, the activation would occur only at high substrate concentrations. It is also possible that the domain interacts with the PHL domain, which is already known to bind PtdIns(3,4,5)P_3_, and hereby reduces the enzyme affinity. The PHL domain showed similar properties to the SH2 domain and negatively impacted efficiency while increasing the overall substrate turnover. The magnitude of the effects appeared to be similar; however, the increase in turnover was more pronounced for the PHL domain, while the reduction in efficiency was more pronounced for the SH2 domain.

**Figure A1:**
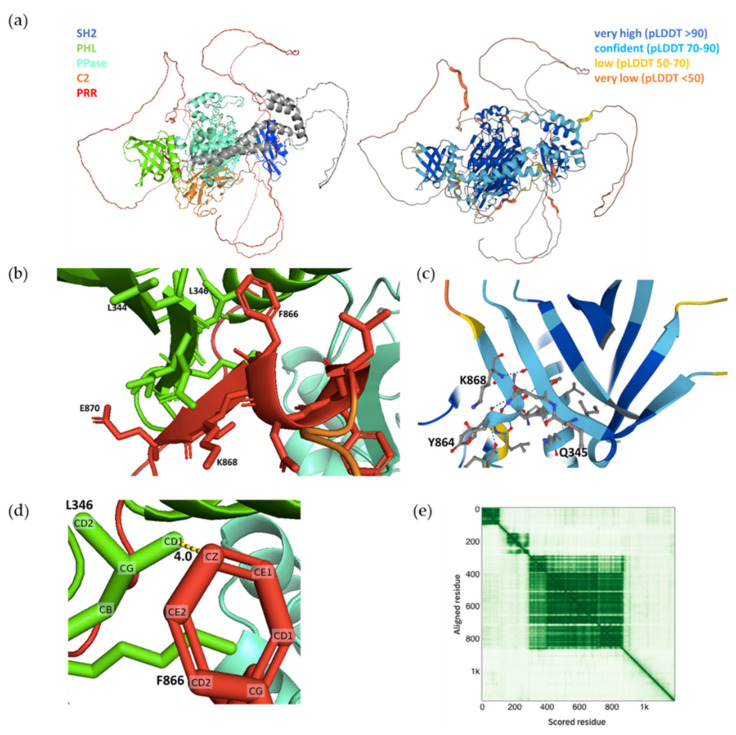
AlphaFold prediction of SHIP1. (**a**) The AlphaFold predicted structure of SHIP1 colored according to the domains in the left panel and the AlphaFold predicted structure of SHIP1 colored according to the model confidence in the right panel. (**b**,**d**) The predicted interface between the PRR and the PHL domain colored according to the domains, and (**c**) the predicted interface colored according to the confidence score. (**e**) The predicted aligned error plot.

We also performed both sigmoidal and hyperbolic regressions for every construct to check for allosteric effects. A Michaelis–Menten graph of an enzyme typically follows the sigmoidal regression curve better than the hyperbolic regression curve if the enzyme is more effective at higher substrate concentrations, indicating its transition to a highly active state by allosteric regulation via its substrate [27]. The R^2^ value, in this case provided by the GraphPad Prism program, is a value with an interval from 0 to 100% and indicates how well the data fit the model applied (sigmoidal or hyperbolic regression). An enzyme with no additional mechanisms regulating its activity should ideally produce strictly hyperbolic curves. In this case, the R^2^ would be higher for the hyperbolic regression than the sigmoidal regression. In our case, however, all constructs followed the sigmoidal regression better than the hyperbolic regression according to the R^2^ values, indicating some regulatory mechanism. Seeing as most variables were controlled in our assay, due to the absence of usual cell contents, we believe that those findings hint towards possible allosteric regulations and allow us to classify SHIP1 as an allosteric enzyme. The reduced difference in the adherence towards the sigmoidal and the hyperbolic regression curves after the truncation of the PRR, the linker, the PHL domain, or the C2 domain suggests that all those domains could take part in the allosteric regulation. This effect was most pronounced for the truncation of the PRR, which may be explained by a PRR to PHL-PPase-C2 interface (Figure A1), and for the truncation of the linker.

**Figure A2:**
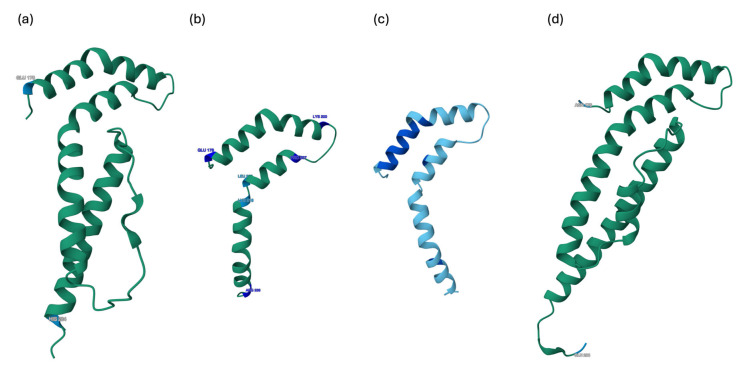
AlphaFold prediction of the possible GTP-bound RhoA-binding domain of SHIP1 and the known GTP-bound RhoA-binding domain of SHIP2. (**a**) Prediction for the entire domain-like structure in SHIP1 in correspondence to (**d**) the full RBD of SHIP2 in the residues ASN176 to GLN298 according to Zhou et al. [29]. (**b**) Part of the possible RBD in SHIP1, which was shown as a sequence homologue in Table A1. (**c**) Part of the possible RBD in SHIP1 as shown in (**c**) but color-coded with respect to the model confidence. Dark blue very high (pLDDT > 90) and light blue high (90 > pLDDT > 70).

The linker between the SH2 domain and the PHL domain surprisingly also influenced the phosphatase activity of the SHIP1 protein. The linker negatively influenced the turnover number but increased the protein’s catalytic efficiency. In particular, the latter was very pronounced. The massive reduction in the K_m_ value, if the linker is expressed as part of the protein, indicates the role of the linker, especially at low substrate concentrations. Previously, the linker has been regarded as a non-important part of the protein, or at least has received no attention in the community. However, in the isoenzyme SHIP2, which has a high sequence homology, with 57.4% identical nucleotides [2], there was a RhoA-binding domain (RBD) proposed to exist in the same linker between the SH2 and PHL domains [29]. The sequence is similar between those two proteins in this region as well. The RhoA-binding domain is proposed to consist of residues 176 to 298 in SHIP2 [29], and this region is similar to residues 186 to 244 in SHIP1, with 44% identities (Table A1). Furthermore, a glance at the predicted protein structure of the SHIP1 enzyme from AlphaFold shows three α-helices starting at GLU178, LYS218, and PRO207 [30,31] (Figure A2). Secondary structures oftentimes hint towards domains in proteins or at least functional areas. This influence of the linker towards the phosphatase activity of SHIP1 in unison with the secondary structure and the similarity to the RhoA-binding domain in SHIP2 now provides the first clue for the existence of a similar domain in SHIP1. However, at this point, this is only a well-founded theory that needs to be proven at a later stage. In general, it can be said that the complementary influence of all the domains gives the full-length protein its specific K_m_ value and affinity towards the substrate, which may allow for negative and positive feedback loops in the signaling pathway, that would otherwise not be possible. A lower K_m_ could lead to overshooting breakdown of PtdIns(3,4,5)P_3_ and therefore of the PI3K/AKT signaling pathway. A higher K_m_ could lead to an abundance and overactivation of the pathway. This could be an interesting pathomechanism that could be explored in the future, which greatly depends on the relevance of the SHIP1 proteins in comparison to other regulators of the same pathway.

All those assessments of the results should be looked at critically, because it is always possible that the mere existence of a domain and several unknown intramolecular interactions leads to a conformational change in the entire enzyme or in parts of the enzyme responsible for the phosphatase activity, either directly or through complex interplay.

Furthermore, the assay is limited to the determination of the activity in solution without membranes or other interacting proteins. It was previously shown that the PHL domain bound to PtdIns(3,4,5)P_3_, which contributed to the membrane localization of SHIP1 [15]. For SHIP1, no allosteric effect of the PHL domain has been shown in the literature before; however, the PHL domain of SHIP2 was recently shown to act as an allosteric activator via the binding of phosphatidylinositol-3,4-bisphosphate (PtdIns(3,4)P_2_) [27]. The SH2 domain and the PRR domain so far have been shown to contribute to membrane recruitment in mammalian cells via protein–protein interactions, but not to protein–lipid interactions or catalysis [14]. Therefore, in the light of both the SH2 and PHL domains contributing to membrane localization, those results do not necessarily represent the activity that may occur under physiological conditions in living cells, where an increase in activity can be achieved only by creating proximity between the enzyme and substrate, or the enzyme and other regulatory proteins and substances. However, it only serves the purpose of determining possible intramolecular interactions and regulations. The C2 domain of SHIP1 was demonstrated to allosterically activate the enzyme by direct association with PtdIns(3,4)P_2_, and this was mediated by K681 [32]. Those phosphoinositide interactions by the PHL and C2 domains were also not investigated in this assay.

**Figure A3:**
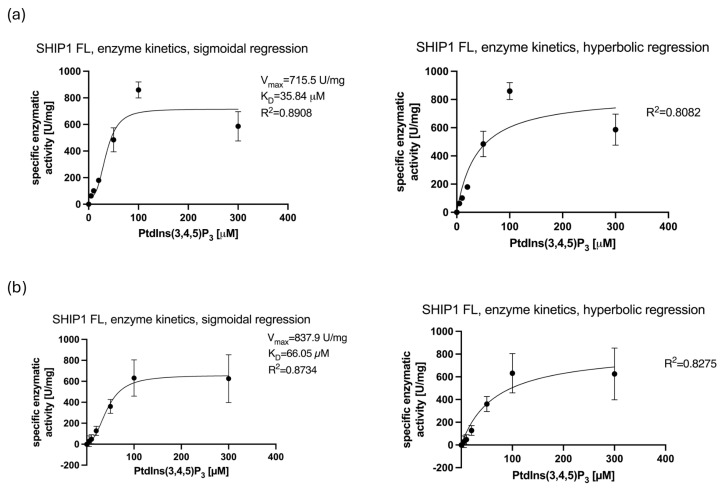
Curves of the Michaelis–Menten equation after the reproduction of the results for SHIP1 FL. (**a**) Determination of the enzyme kinetics of SHIP1 FL towards PtdIns(3,4,5)P_3_. The specific activity of SHIP1 FL was determined for substrate concentrations ranging from 0 to 300 µM, plotted against the substrate concentration in µM and analyzed by sigmoidal or hyperbolic regression to determine the V_max_ and K_m_. Error bars indicate standard deviations. *n* = 3. (**b**) Reproduction of the results for the enzyme kinetics of SHIP1 FL. The specific activity of SHIP1 FL was determined for substrate concentrations ranging from 0 to 300 µM, plotted against the substrate concentration in µM and analyzed by sigmoidal or hyperbolic regression to determine the V_max_ and K_m_. Error bars indicate standard deviations. *n* = 3.

Also, one problem that arose was the fragmentation of the protein, as can be seen in the SDS-PAGE after Coomassie staining (Figure 2a,c). In general, more rapid protein degradation of truncation mutants has previously been reported in the literature [33]. We tried to limit the degradation and fragmentation of the protein as much as possible with the addition of protease inhibitors, reduced lysis times, a cool working environment of never more than 4 °C, and ice cooling whenever possible, as well as the immediate use of the purified protein without freezing and thawing. Still, we were not successful in generating better SDS-PAGE results. The additional bands do not seem to be impurities, because the bands could be detected in the Western blot by the specific Strep-MAB classic antibody (IBA) (Figure 2b,d). Initially, we were concerned that the degradation could interfere within the assay. The whole process, however, was reproduced by different people with similar results for the full-length construct (Figure A3), indicating that this method is still sufficient for the analysis to the extent we wanted and presented in this paper. The small variance in results can be attributed most likely to interpersonal differences in, for example, pipetting technique and SDS-PAGE interpretation. One other factor for the higher activity of SHIP1 FL in the second dataset (Figure A3b) is probably due to the fact that the protein was used immediately after the purification. We oftentimes observed that even one freeze-and-thaw process was enough to reduce a protein’s activity.

When considering the predictions made by AlphaFold, it must be acknowledged that AlphaFold is an artificial intelligence (AI) program trained on existing data, which means that the prediction is only as good as the training data that were used. This implies that the predictions are most accurate for well-defined and often otherwise analyzed structures, such as SH2 domains. The prediction becomes less trustworthy if dealing with more uncommon structures, like the proline-rich region (Figure A1). Rho-A-binding domains such as in the Serine/threonine-protein kinase N1 (PKN1), however, are generally well-defined from X-ray crystallography and nuclear magnetic resonance (NMR) spectroscopy structure predictions [34], which means the AlphaFold model should have enough data to accurately determine the structure of domains with similar amino-acid sequences, such as the possible Rho-A-binding domain in SHIP1 (Figure A2). Notably, the AlphaFold2 model has been reported to be even more accurate than protein predictions from NMR spectroscopy after comparing 904 different human proteins with regard to the Analyzer of Structural Unraveled Residues and Residue (ANSURR) score [35]. The experimental validation of the same domain in the isoenzyme SHIP2 [29] further increases our confidence.

The final topic of discussion focuses on comparing our findings with those currently available in the literature. We observed some discrepancies between our findings and those reported by Bradshaw et al. on the K_m_ and k_cat_ values for the SHIP1 constructs SHIP1 5PPase and SHIP1 5PPase-C2. They calculated significantly lower values for the catalytic parameters [28]. Those differences can likely be attributed to variations in the experimental conditions employed. One significant factor was the differences in electrolyte concentrations, particularly potassium, which is known to influence enzymatic activity [36,37]. Another important aspect to consider is the temperature at which the assays were performed. In our experiments, we performed the assays at 37 °C, aligning closely with the conditions within human biological systems, while Bradshaw et al. chose to conduct their assays at room temperature, resulting in lower values for the temperature-dependent catalytic parameters. However, the general findings are consistent with each other. Bradshaw et al. also found the C2 domain to positively affect substrate turnover [28]. The C2 domain in SHIP1 has been known to be required for allosteric activation through positive feedback from its end-product PtdIns(3,4)P_2_ [17,38]. They also reported a lack of conformity to Michaelis–Menten kinetics in accordance with our findings, regarding the better fit to a sigmoid regression model [28]. Additionally, another study demonstrated an inhibitory role of the SH2 domain, and we similarly noted an increase in catalytic activity with our SH2 truncation mutant, indicating a negative impact of the SH2 domain on the catalytic efficiency [39].

## 5. Conclusions

**Figure 4:**
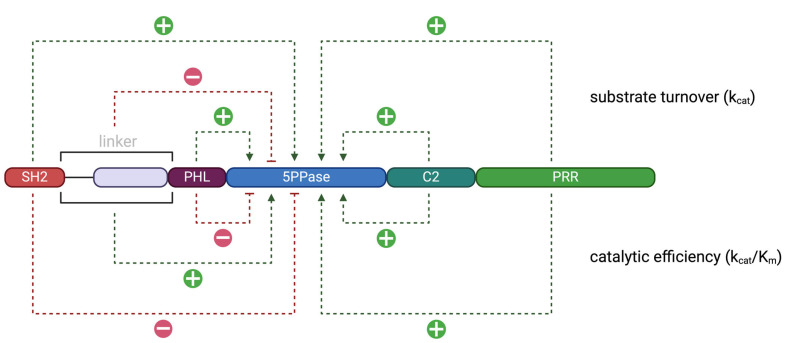
Model of the SHIP1 domains and their impact on catalytic parameters [Created in BioRender. Müller, S. (2023) https://BioRender.com/j86a434, (accessed on 1 November 2024)]. The purple depicted region of the linker is the possible GTP-bound RhoA-binding domain.

**Figure 5:**
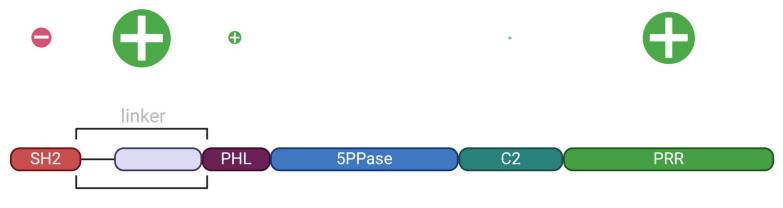
Model of the SHIP1 Domains and Their Impact on Allosteric Regulation of the Catalytic Activity [Created in BioRender. Müller, S. (2024) https://BioRender.com/q81p221, (accessed on 1 November 2024)]. The plus and minus signs represent the change in ΔR^2^ = R^2^_sig_ − R^2^_hyp_, which is the difference in coefficient of determination (R^2^) for the sigmoidal regression (sig) and the hyperbolic regression (hyp), when the corresponding domain is added to a SHIP1 construct. A plus sign represents an increase in the adherence to the sigmoidal regression compared to the hyperbolic regression. The plus and minus signs are scaled to the magnitude of the effect. SH2 − 2.075%; linker + 6.04%; PHL + 1.44%; C2 + 0.36%; PRR + 5.445%.

In conclusion, all the domains of the SHIP1 protein were found to exhibit some form of influence on the catalysis of the protein (Figure 4). The full-length protein showed the highest substrate turnover number out of all constructs, indicating that additional domains to those directly involved in phosphoinositide binding may contribute to full enzymatic activity. The SH2 domain increased substrate turnover but negatively affected catalytic efficiency. The linker between the SH2 domain negatively influenced the turnover number but increased the protein’s catalytic efficiency. The PHL domain showed similar properties as the SH2 domain and negatively impacted efficiency while increasing the overall substrate turnover to a similar extent. The C2 domain as well as the PRR increased both substrate turnover and catalytic efficiency. The regression analysis of the Michaelis–Menten graph showed SHIP1 to be an allosteric enzyme, with the PRR and the linker being the most involved domains, but the PHL and C2 domains seem to also contribute in that regard (Figure 5). In particular, the large significance of the linker between the SH2 and PHL domains in the regulation of the protein’s activity, as well as the high sequence homology of one part of the linker to other known Rho-A interaction motifs, suggests that one part of the linker constitutes a not-yet-described RhoA-binding domain.

## Figures and Tables

**Table 1:** Primer list.

Primer Name	Primer Sequence
SHIP1_FL_fw	5′-AGCGGCTCTTC**AATG**GTCCCCTGCTGGAACCATG-3′
SHIP1_FL_rv	5′-AGCGGCTCTTC**TCCC**CTGCATGGCAGTCCTGCC-3′
SHIP1_RBD_(N)_fw	5′-AGCGGCTCTTC**AATG**CCAGAAGAGCATCTTAAGGCCATCCAA-3′
SHIP1_RBD(C)_fw	5′-AGCGGCTCTTC**AATG**GACCAGCAGCTCTCCCCG-3′
SHIP1_PHL_fw	5′-AGCGGCTCTTC**AATG**TCCCTTATCCCTCCAGTCAC-3′
SHIP1_PHL_rv	5′-AGCGGCTCTTC**TCCC**CATGTCGGGCTCCGGCTG-3′
SHIP1_C2_fw	5′-AGCGGCTCTTC**AATG**GCAGGAGTCACTTCCCAGT-3′
SHIP1_C2_rv	5′-AGCGGCTCTTC**TCCC**CTCCCTCGTCTTGCCCTG-3′
SHIP1_5PPase_fw	5′-AGCGGCTCTTC**AATG**CCGGAGCCCGACATGATC-3′
SHIP1_5PPase_rv	5′-AGCGGCTCTTC**TCCC**AGTGACTCCTGCCTCAAATG-3′

The table shows all the specific primers used for the Stargate^®^ cloning protocol. Underlined sections represent the *Lgu*I recognition site, whereas the bold sections represent the combinatorial sites. The orientation of the primers allows for a unidirectional insertion of the DNA into the vector.

**Table 2:** Protein concentration after size-exclusion chromatography.

	FL	5PPase	PHL-5PPase	5PPase-C2	PHL-5PPase-C2	ΔSH2	ΔSH2-Linker	ΔPRR
concentration [ng/µL]	537.78	1034.67	604.45	1190.25	634.92	827.20	643.32	260.40

This table shows the achieved concentrations of the SHIP1 domain constructs in ng/µL after size-exclusion chromatography and prior to dilution to 5 ng/µL for the phosphatase assay.

**Table 3:** Approximate molecular weight of SHIP1 domain constructs.

	FL	5PPase	PHL-5PPase	5PPase-C2	PHL-5PPase-C2	ΔSH2	ΔSH2-Linker	ΔPRR
molecular weight [kDa]	145	36	48	52.5	66	123	102	100

This table shows the approximate molecular weight of the SHIP1 domain constructs in kDa (=kg/mol) according to the ExPASy Compute Pi/Mw tool and the established molecular weight of the 145-kDa-SHIP1 (SHIP1_FL).

**Table 4:** Comparison of the enzyme kinetic parameters of the SHIP1 constructs.

	FL	5PPase	PHL-5PPase	5PPase-C2	PHL-5PPase-C2	ΔSH2	ΔSH2-Linker	ΔPRR
V_max_ [U/mg]	715.5	513.6	n.d.	493.6	942.2	481.3	835.4	876.9
K_m_ [µM]	35.84	293.7	n.d.	27.6	105.7	13.45	123.9	52.44
k_cat_ [s^−1^]	1729.13	308.16	n.d.	431.90	1036.42	989.42	1423.71	1456.30
k_cat_/K_m_ [L/(mol × s)]	4.82 × 10^7^	1.05 × 10^6^	n.d.	1.56 × 10^7^	9.81 × 10^6^	7.36 × 10^7^	1.15 × 10^7^	2.78 × 10^7^

The table shows V_max_, K_m_, k_cat_ and k_cat_/K_m_ of SHIP1 FL, SHIP1 5PPase, SHIP1 PHL-5PPase, SHIP1 5PPase-C2, SHIP1 PHL-5PPase-C2, SHIP1 linker-PHL-5PPase-C2-PRR (ΔSH2), SHIP1 PHL-5PPase-C2-PRR (ΔSH2-linker) and SHIP1 SH2-linker-PHL-5PPase-C2 (ΔPRR); n.d. = not de-terminable; *n* = 3.

**Table 5:** Adherence to the regression model of the SHIP1 constructs.

	FL_average_	5PPase	PHL-5PPase	5PPase-C2	PHL-5PPase-C2	ΔSH2	ΔSH2-Linker	ΔPRR
R^2^_sig_	0.8821	0.9794	n.c.	0.5862	0.853	0.8242	0.8264	0.9686
R^2^_hyp_	0.81785	0.9725	n.c.	0.5757	0.8281	0.7392	0.8018	0.9588
R^2^_sig_/R^2^_hyp_	1.0786	1.0071	n.c.	1.0182	1.0300	1.115	1.0307	1.010
R^2^_sig_ − R^2^_hyp_	0.06425	0.0069	n.c.	0.0105	0.0249	0.085	0.0246	0.0098

This table shows the adherence towards the sigmoidal or hyperbolic regression model, expressed by the coefficient of determination R^2^. R^2^_sig_ is the coefficient of determination for the sigmoidal regression and R^2^_hyp_ is the coefficient of determination for the hyperbolic regression model. The values were generated using the GraphPad Prism program. The values for the PHL-5PPase constructs were not calculated (n.c.) due to the not-determinable catalytic parameters for this construct.

## Data Availability

The raw data supporting the conclusions of this article will be made available by the corresponding author on request.

## Appendix A

Aiming for a possible explanation for the increased catalytic efficiency of SHIP1 FL compared to SHIP1 PHL-PPase-C2, before knowing the individual contribution of the SH2 domain and PRR to catalysis, the structure of SHIP1 FL was predicted using AlphaFold [30,31] (Figure A1). We chose to use AlphaFold because, within recent years, the program has become more recognized in the scientific community, and there is only one nuclear magnetic resonance spectroscopy (NMR) structure in the SH2 domain of SHIP1 [40]. Moreover, there are several X-ray structures of its 5PPase and C2 domains in a complex with other compounds [28], but there are still no experimental structure analyses of the full-length SHIP1 protein. In general, structure prediction in AlphaFold was of high confidence (pLDDT > 70), with the exception of most of the PRR, indicating that this might be flexible, as has been described for proline-rich domains before [41] (Figure A1a). According to the predicted aligned error plot aligning to the core region of PHL-PPase-C2, a small portion at the beginning of the PRR around amino acid 900 with a low expected position error was identified, which could be an indication to a possible interdomain interaction between this section of the PRR and the phosphoinositide-binding core (Figure A1e). We were able to identify a short β -sheet at the beginning of the PRR (L863-E870), which was predicted with a high confidence score and may interact with a second short β-sheet within the PHL domain (L341-S349) (Figure A1b,c). A hydrophobic interaction between F866 and L346 may likely occur from the distance measurements (Figure A1d). In order to verify the predicted putative positive effect of the PRR via a PRR to PHL-PPase-C2 interface, this remains one possible target for future mutagenesis studies. This further supports our findings regarding the PRR and their positive influence on catalytic efficiency as well as substrate turnover, and could also be one explanation for the massively reduced delta of adherence to the regression models after its truncation.

**Table A1:** Protein blast of the proposed RhoA-binding domain of SHIP2 and the sequence of the SHIP1 protein.

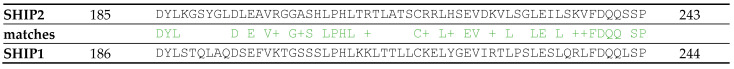

This table shows a protein blast of the proposed RhoA-binding domain of SHIP2, in the residues 176 to 298, against the protein sequence of SHIP1 in the bottom row. The sequence homology starts at the ninth residue of the proposed RBD and in residue 185 in the SHIP2 sequence. The matches are shown in the middle in green. Overall, 26 out of 59 residues were identical (44%), and 34 out of the 59 were detected as positives (57%). Zero gaps (0%) were detected. The blast was performed with the protein blast tool from NCBI at https://blast.ncbi.nlm.nih.gov/Blast.cgi, accessed on 1 November 2024.
